# Anti-CD20 therapy in lupus nephritis: A revisit

**DOI:** 10.1515/rir-2025-0009

**Published:** 2025-07-01

**Authors:** Chi Chiu Mok

**Affiliations:** Department of Medicine, Tuen Mun Hospital, Hong Kong SAR, China

Renal disease is one of the most common and serious manifestations of systemic lupus erythematosus (SLE), particularly in the Asians and African Americans.^[1]^ According to a study published in 2013, the life expectancy of having a diagnosis of SLE in women was reduced by 20 years compared to the general population.^[2]^ The standardized mortality ratio (SMR) of southern Chinese patients with non-renal SLE was 4.8, but increased to 9.0 for having kidney involvement.^[3]^ Despite a meta-analysis demonstrated the 5-year incidence of end-stage kidney disease in SLE has reduced from 16% to 11% from the 1970 s’ to 1990 s’, further improvement was not sustained after the mid-1990 s’.^[4]^ Thus, there is an unmet need for novel therapeutic agents or strategies to improve the treatment response and prognosis of lupus nephritis (LN).^[5]^

B cells are pivotal in the pathogenesis of SLE because they produce autoantibodies that mediate inflammation and tissue damage. B cells are also capable of presenting self-antigens to activate T cells and producing proinflammatory cytokines. ^[6]^ Subpopulations of immature B cells, termed transitional B cells, which are dependent on B cell activation factor (BAFF) for maturation to immunocompetence, and autoreactive age-associated B cells are expanded in SLE patients, whereas the interleukin-10 (IL-10) producing regulatory B cells are defective.^[7]^ Eliminating the B cells, especially the autoreactive clones and reconstituting the B cell repertoire, is a major strategy for the treatment of SLE.^[8]^ Following the initial success in rheumatoid arthritis,^[9]^ B cell depletion by the chimeric monoclonal antibody, rituximab, started to be explored in SLE patients.^[10]^ Rituximab depletes B cells that express CD20, from the stage of pre-B to mature B cells, including the memory B cells and plasmablasts.^[6]^ Marrow pro-B cells and terminally differentiated plasma cells are spared. Two randomized controlled trials (RCTs) of rituximab in extra-renal and renal SLE were performed but the primary efficacy end points were not achieved.^[11,12]^ In the LN study (LUNAR),^[12]^ two courses of rituximab (1 g 2-weekly for two doses; 6 months apart) led to greater improvement in anti-dsDNA and complement levels compared to placebo (PBO), but did not result in a significantly better rate of complete renal response (CRR) at week 52 when combined with the standard of care (SOC) of glucocorticoids (GCs) and mycophenolate mofetil (MMF).^[7]^ Despite the futility of these RCTs, rituximab continued to be used “off-label” in the treatment of SLE. Data from case series and registries supported the efficacy of rituximab in refractory SLE manifestations, including LN, with up to two-thirds of patients having a clinical response,^[13]^ although the retrospective nature of these data is subject to bias towards efficacy due to the lack of a controlled group. International guidelines also recommend the use of rituximab in refractory SLE and LN.^[14, 15, 16, 17]^

Ocrelizumab is a fully humanized anti-CD20 monoclonal antibody with enhanced antibody-dependent cell-mediated cytotoxicity (ADCC) and reduced complement-dependent cytotoxicity (CDC) compared to rituximab.^[18]^ In the phase III RCT (BELONG),^[19]^ which had a similar study design and inclusion criteria with the LUNAR study,^[12]^ the renal response rate in those who completed 32 weeks’ treatment was not significantly higher in the ocrelizumab than the PBO group when combined with GCs and either MMF or low-dose cyclophosphamide. Moreover, the study was prematurely terminated because of the concern of serious infections.

Ofatumumab is a human monoclonal antibody that directs against a distinct extracellular epitope of CD20 on B cells. Compared to rituximab, ofatumumab has a slower dissociation kinetics from its target and is a more potent activator of CDC.^[20]^ As a result, the potency of B cell depletion is enhanced. In a single arm study of LN, ofatumumab has been shown to deplete B cells with comparable efficacy and reconstitution kinetics to rituximab.^[21]^ Efficacy of ofatumumab was reported in case series of LN.^[21, 22, 23]^ However, no RCTs are pursued in SLE or LN.

Obinutuzumab is a recombinant humanized type II anti-CD20 monoclonal antibody that is glyco-engineered for greater affinity for the FcγRIII on effector cells, leading to enhanced ADCC, direct B-cell killing, and less reliance on CDC.^[24]^ Obinutuzumab has been shown to be more effective than rituximab in the depletion of B cells, activation of natural killer cells, and amelioration of nephritis in murine lupus.^[25]^ A phase II PBO-controlled RCT of obinutuzumab (NOBILITY) in 125 patients with LN (class III/V) treated with background GC/MMF demonstrated superiority of the biologic to PBO in achieving CRR at week 52 through 104.^[26]^ Obinutuzumab was not associated with an increased serious infections or deaths. Post-hoc analyses demonstrated this biologic reduced the risk of LN flares, first estimated glomerular filtration rate (eGFR) decline by 30% or 40%, and a composite outcome of treatment failure, doubling of serum creatinine, or death as compared to PBO.^[27]^ These results are promising and show efficacy of anti-CD20 therapy in the preservation of kidney function in LN. The phase III RCT of obinutuzumab (REGENCY) ^[28]^ further consolidated the results of the NOBILITY trial.^[26]^ In this study, 271 patients with active LN (class III/IV±V) were randomized to obinutuzumab (1 g 2-weekly for 2 doses and repeat at week 24) or PBO in addition to GC/MMF. The primary outcome, CRR rate at week 76, was significantly higher in the treatment than PBO group (46% vs. 33%). Secondary end points such as CRR together with a maintenance prednisone dose of ≤7.5 mg/day between weeks 64 and 76 and a urinary protein-to-creatinine (uP/Cr) ratio < 0.8 were also in favor of obinutuzumab. However, serious adverse effects and infections were more frequent in obinutuzumab-treated patients, which were driven by an increased incidence of upper respiratory infections, coronavirus disease 2019 (COVID19)-related pneumonia and other infections. Greater adjusted between-group differences in CRR rate were observed in those patients with higher serological SLE activity, uP/Cr > 3.0 and histological class IV±V disease.

So, why is there a discrepancy of results among the older and newer generation anti-CD20 biologic trials in LN? Criticisms of the LUNAR study ^[12]^ included an inadequate duration of observation (52 weeks) as the primary outcome and the lack of power to detect a difference in partial renal response (PRR) between rituximab and PBO.^[29]^ In fact, at week 78, an exploratory end point, the proportion of patients achieving CRR or PRR together was significantly higher in the rituximab than PBO group (74% vs. 57%), indicating improvement in renal parameters might be delayed with anti-CD20 therapy. In the absence of repeat renal biopsy data, it is uncertain if some of the patients with PRR (but not CRR) might have actually achieved histological remission at week 52. There are several other factors that may affect the efficacy of the anti-CD20 biologics in SLE. The FcγRIIIa genotypes contributed to the variability of B cell depletion efficacy by rituximab ^[30]^ and it is not certain how the genotype distribution of the participants in the LUNAR study ^[12]^ might have influenced the primary outcome. The development of neutralizing antibodies to the chimeric compound might also undermine the durability of B cell depletion.^[31]^ Finally, the failure of depletion of pathological B cells in tissues may contribute to treatment failure and disease relapse in some SLE patients.^[7]^ This is supported by the observation that with more profound depletion of the CD19^+^ B cells by chimeric antigen receptor T cell therapy (CAR-T), the efficacy in SLE could be prolonged.^[32]^

In the aborted ocrelizumab LN study,^[19]^ the unbalanced rate of serious infections, particularly in Asian study sites, was partly attributable to the high dose of MMF (3 g/day; not tapered until week 48), regardless of body weight (which was lower in Asians), adopted in the protocol. The obinutuzumab LN RCTs have taken into account the above caveats in study design. Participants received protocol-based oral prednisone of 0.5 mg/kg/day with the goal to taper to 7.5 mg/day by week 12 and 5 mg/day by week 24, and the dose of MMF dose was targeted to 2.0–2.5 g/day.^[28]^ The lower doses of GC/MMF might help to reduce the overall risk of infective complications and the assessment of primary efficacy was performed at week 76 instead of week 52. The higher potency of obinutuzumab in B cell depletion, the longer observation period and the minimization of background immunosuppression might have contributed to the better results in LN. As repeat renal biopsy was not part of the study design in the anti-CD20 LN RCTs,^[12,26,28]^ it remains unclear if obinutuzumab is more effective than rituximab in depleting the disease promoting B cells in the kidneys. Moreover, compared to the phase II RCT ^[26]^ in which there was no increased incidence of serious infections with obinutuzumab, more infective episodes were gauged as serious adverse events in the treatment arm of the phase III study,^[28]^ which was caused by viruses (including COVID-19) and other micro-organisms. Thus, caution must be exhibited for the use of obinutuzumab in SLE patients who are particularly susceptible to infective complications. Vaccination against common infections and appropriate antimicrobial prophylaxis according to local guidelines is deemed necessary.^[33]^

Recent RCTs have shown a combination of belimumab,^[34]^ calcineurin inhibitors (CNIs)^[35,36]^ and anti-CD20 biologic ^[28]^ with SOC resulted in a higher renal response rate than SOC alone in LN. This leads to the recommendation for upfront combination therapy of LN by international guidelines, particularly in patients at higher risk of renal progression, in order to maximize the treatment response.^[14,16,17,37]^ However, there are issues of cost-effectiveness in less affluent countries and safety in susceptible patients at risk of serious infective complications with this treatment approach.^[38]^ The choice among anti-CD20, anti-BAFF and the CNIs should be individualized, taking into consideration the clinical characteristics of patients, medical comorbidities, renal function, histological features, past treatment response of LN, as well as the expected tolerability and compliance, and the wish of patients for conception. Anti-CD20 and the CNIs appear to have better efficacy than anti-BAFF in patients with more severe proteinuria according to subgroup data of the pivotal RCTs. While the CNIs may work better in patients with histological evidence of podocytopathy, they are difficult to titrate in patients with impaired renal function.^[39]^ Despite the frequent use of rituximab for treating refractory non-renal SLE, the pivotal RCT (EXPLORER) did not show superiority of rituximab over PBO.^[11]^ Based on the paucity of data regarding the efficacy of obinutuzumab and the CNIs in extra-renal SLE, belimumab may be a better option in patients with both renal and extra-renal activity. Finally, the use of rituximab biosimilars is common in the Asia Pacific region and the Asian consensus recommends the biosimilars as alternatives for the bio-original products.^[17]^ It remains debatable, of course, if obinutuzumab should be replaced by the rituximab biosimilars for the initial or rescue therapy for LN in Asian countries due to the cost concern. Nevertheless, with the availability of more novel therapies for LN,^[40]^ including the newer generation anti-CD20 agents, it is hoped that the outcome and quality of life of patients with LN will further improve in the near future.
